# Synergic Enzymatic Strategy for Simultaneous Cello-/Xylo-Oligosaccharide Production from Sugarcane Straw via a Novel One-Step Protic Ionic Liquid Delignification–Deacetylation Pretreatment

**DOI:** 10.1021/acsomega.5c12305

**Published:** 2026-07-01

**Authors:** Igor S. Gonçalves, Rosana Goldbeck, Telma T. Franco, Marcus Bruno S. Forte

**Affiliations:** † Bioprocess and Metabolic Engineering Laboratory, Department of Food Engineering and Technology, Faculty of Food Engineering, Universidade Estadual de Campinas (UNICAMP), Rua Monteiro Lobato, 80, Zeferino Vaz, Campinas, São Paulo 13083-862, Brazil; ‡ Faculty of Chemical Engineering, Universidade Estadual de Campinas (UNICAMP), Rua Albert Einstein, 500, Zeferino Vaz, Campinas, São Paulo 13083-852, Brazil

## Abstract

Cello- (COS) and xylo-oligosaccharides (XOS) are value-added carbohydrates obtained from lignocellulosic biomasses and studied due to their potential in the biotechnology industry. Our study demonstrates an efficient and integrated strategy for the simultaneous production of COS and XOS using sugarcane straw that was previously delignified and deacetylated through a one-step pretreatment with a mixture of protic ionic liquids (2-hydroxyethylammonium acetate [Mea]­[Ac] and hexanoate [Mea]­[Hex]). Under improved experimental design conditions (40 °C, cellulase 50 FPU g_biomass_^–1^, and xylanase 30 U g_biomass_^–1^), the process achieved remarkable yields within just 3 h: 77.66 mgCOS g_biomass_^–1^ (productivity of 25.89 mgCOS g_biomass_^–1^ h^–1^ and specific productivity of 0.52 mgCOS FPU^–1^ h^–1^) and 19.27 mgXOS g_biomass_^–1^ (productivity of 6.42 mgXOS g_biomass_^–1^ h^–1^ and specific productivity of 0.21 mgXOS U^1–^ h^–1^). Results showed that milder temperatures and shorter times favor enzymatic hydrolysis for producing oligosaccharides, and the presence of xylanase is crucial for synergistic action and for the simultaneous obtaining of COS and XOS, making the enzymatic route more economical for obtaining bioproducts. Thus, the use of ionic liquids as pretreatment agents, a highly sustainable and environmentally friendly process, promotes the valorization of lignocellulosic waste, such as sugarcane straw, and enables the production of high value-added biomolecules, such as oligosaccharides.

## Graphical abstract

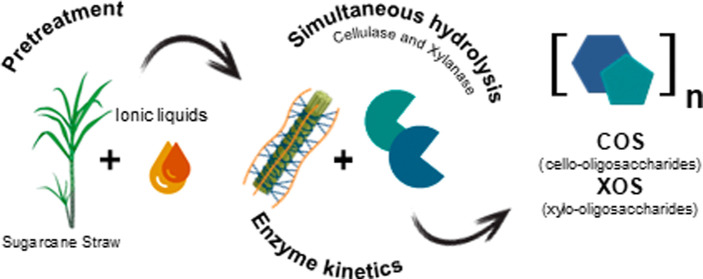

## 1 Introduction

Oligosaccharides are carbohydrates that make up a small number of sugar molecules linked together and can be formed from 2 to 10 monosaccharides joined by glycosidic bonds. These biocomponents have aroused interest in the food industry regarding their incorporation in products for preparing functional foods and can be obtained chemically or biologically.^1,2^ Generally, the chemical route uses acids or bases for material degradation and has the disadvantage of high toxicity, equipment corrosivity, and the use of reagents for neutralization and degradation of the components into monosaccharides and is an uninteresting process to obtain oligosaccharides. Enzymes, the biological sources, are widely used to obtain specific products due to their specificity and selectivity in breaking bonds and obtaining desirable components. They can be used freely and also immobilized for later reuse and are nontoxic, which contribute to future applications of the biocomponent in the preparation of food and pharmaceutical products.^3^ Cello-oligosaccharides and xylo-oligosaccharides are classified as oligosaccharides which come from the cellulosic and hemicellulosic fractions of biomass, respectively. Cello-oligosaccharides (COS) consist of repeating glucose units linked to each other by β-1,4 glycosidic bonds, whereas xylo-oligosaccharides (XOS) are composed of xylose units, also connected through β-1,4 glycosidic bonds. In both cases, the oligosaccharides are classified according to their degree of polymerization.^1,4,5^

COS and XOS are molecules exploited in studies as prebiotics in human and animal food with benefits for the gastrointestinal system,^6–11^ and in fermentation processes, it is relevant in the use of modified microorganisms that consume these oligos and thus reduce the contamination of the process by other glucose-/xylose-consuming organisms.^4,12,13^ Previous studies have evaluated the release of COS and XOS separately from different biomasses subjected to pretreatments using robust enzyme cocktails and/or mixtures of purified enzymes, as well as purified biomass substrates.^3,12,13^ XOS production has also been investigated from purified arabinoxylan using purified enzymes,^14^ and enzyme mixtures have been applied for hemicellulose depolymerization.^15^ Various biomasses have been explored for COS and XOS production, including sugarcane bagasse and bamboo culm for XOS generation via enzymatic hydrolysis;^12^ sugarcane straw pretreated with ionic liquids and under hydrothermal conditions;^4^ and sugarcane straw and coffee husk subjected to alkaline hydrothermal pretreatment for COS production.^1^ These studies typically employ long enzymatic hydrolysis times (24 h and/or 48 h), in addition to the use of purified enzymes and/or purified substrates, with a focus on obtaining a single bioproduct. This approach results in low-productivity processes, high operational costs, and greater complexity due to additional purification steps. In addition, the need for high-purity inputs compromises the economic viability on an industrial scale, reducing the applicability of the process in biorefinery contexts, where the integrated production of multiple value-added compounds would be more advantageous. The lack of full use of biomass also represents a limitation in terms of sustainability and overall process efficiency.

Several biomasses are used to obtain oligosaccharides, and in Brazil, sugarcane straw is vastly abundant,^17,18^ making it a viable source of biomass for bioproduct conversion.^5^ Its features allow it to be exploited to obtain its fractions: cellulose, hemicellulose, and lignin, with the potential for elaborating various products.^4,5,12,13,18^ Straw use helps to value the waste, since its final destination would be burning to generate energy in the form of heat or improper disposal, thus contributing to sustainability in industrial processes and reduction of gas emissions into the environment.^5,18^

Use of enzymes in the production of oligosaccharides from lignocellulosic (LC) biomasses requires a previous step called pretreatment, which consists of the deconstruction of the rigid matrix and thus facilitates their accessibility for the release of desirable components. Use of protic ionic liquids in pretreatments has stood out compared with other methods due to their favorable characteristics such as the ability to make specific adjustments designed to meet the needs of the pretreatment, high selectivity in the dissolution of LC components, recycling and reuse in the process, and comparable hydrolytic efficiency even after pretreatment cycles, reducing costs and environmental impact.^5,18,19^

After reducing the recalcitrance of the LC matrix, the next step is enzyme application. There are several enzymes used that can act separately or synergistically to improve the overall reaction efficiency, increasing the process yield. Combining enzymes enables flexibility and adaptability in different substrates under varying conditions and allows to optimize the production process by adjusting the enzyme load of each enzyme to meet the needs of the reaction.^20,21^ Studies have reported that the mixture of specific and/or purified enzymes has favored the yield of bioproducts from biomass, as well as the synergy of commercial cocktails in COS or XOS production from sugarcane straw and bagasse, resulting in process improvement and economy;^5,14^ however, they use purified enzymes and/or long times (48–72 h), which make the process unfeasible, since 20 to 30% of the process costs are attributed to enzymatic hydrolysis, making it more expensive and with low productivity.^3^ Studies explore the production of COS and XOS separately and employ fractionation or purification steps to isolate cellulose and hemicellulose prior to enzymatic hydrolysis (Table 1). The reported strategies involve multistage processing, including pretreatment, solid–liquid separation, and selective enzymatic hydrolysis of individual biomass fractions.^4,5,12,13^ Such fractionation steps not only increase process complexity but also lead to higher operational costs and longer processing times.

**Table 1:** Pretreatment and Enzymatic Hydrolysis Strategies for the Production of COS and XOS from Lignocellulosic Biomass

substrate	pretreatment	enzymatic system	hydrolysis time (h)	COS + XOS	mg/gsubstrate
sugarcane straw^4^	hydrothermal	endoglucanases, LPMOs, CDH, and different additives	48	COS	60.7
sugarcane straw^5^	protic ionic liquid	celluclast 1.5 L	6	COS	102.2
sugarcane straw^1^	hidrotérmico alcalino e purificação da cellulose	endoglucanase, exoglucanase, and feruloyl esterase	48	COS	63.56
bamboo culm^12^	alkaline extraction and purified hemicellulose	Shearzyme	24	XOS	250.0
sugarcane straw^13^	alkaline extraction and purified holocellulose	multiple-stage cellulase 1,4-β-endoglucanase	6 + 18 + 24	COS	85.43
coffee husk^13^	alkaline extraction and purified holocellulose	multiple-stage cellulase 1,4-β-endoglucanase	6 + 18 + 24	COS	60.36
sugarcane straw^14^	alkaline extraction and purified hemicellulose	endoxylanase, α-L-arabinofuranosidase, feruloyl esterase	48	XOS	172.5
coffee husk^14^	alkaline extraction and purified hemicellulose	endoxylanase, α-L-arabinofuranosidase, feruloyl esterase	48	XOS	152.5
sugarcane bagasse^16^	alkaline treatment and acid precipitation	Shearzyme	24	XOS	225.0

Enzyme load and temperature are variables that can have a significant impact on the efficiency of enzymatic reactions.^20,21^ Studying different enzyme loads and temperatures can affect the yield of COS and XOS, and the combination of these variables can optimize the process for the simultaneous obtaining of components. Studies in the literature explore obtaining COS and XOS separately, and we know that enzymes are used to obtain sugar monomers for fermentation purposes;^4,5,12,13^ however, no studies were found in the literature on the use of enzymatic mixtures for the simultaneous production of COS and XOS from a single biomass, without the need for purification.

This research brings an optimization of the release of COS and XOS using cellulase and xylanase loads and different enzymatic hydrolysis temperatures, evaluating the release time of these oligosaccharides and improving enzymatic mixtures to obtain COS and XOS from sugarcane straw biomass, previously delignified and deacetylated through a one-step pretreatment with a mixture of protic ionic liquids. First, we evaluated the release of COS and XOS individually by using cellulase and xylanase, respectively. After selecting the pretreated biomass (sugarcane straw), an experimental design investigated the mixture of different cellulase and xylanase enzymatic loads and temperatures in the simultaneous release of COS and XOS combined with different hydrolysis times. As the best process conditions were determined, validation was performed to confirm that the process is reliable, repeatable, and controlled. Answers were evaluated regarding the total release of COS and XOS, productivity, and specific productivity, aiming at the optimization and reduction of time and costs of enzymatic hydrolysis, important factors in applications that use biological molecules such as enzymes. Obtaining two value-added compounds in a single process also improves economic viability and expands commercial applications, due to the functional properties of COS and XOS, and producing these biomolecules simultaneously is a smart and economically attractive technological strategy for biorefineries, as it maximizes the use of biomass, adds value, and promotes industrial sustainability.

## 2 Materials and Methods

### 2.1 Materials

The reagents used in the synthesis of ionic liquids were acetic acid (99.7% pure, Synth, Brazil), hexanoic acid (99% pure, Sigma-Aldrich), and ethanolamine (99% pure, Dinâmica, Brazil). 2-Hydroxyethylammonium acetate ([Mea]­[Ac]) and 2-hydroxyethylammonium hexanoate ([Mea]­[Hex]) were synthesized using the acid–base neutralization method.^5^ The enzymes used were the enzyme cocktails Novozym 50,013 and Shearzyme 500 L; and citric acid (99% pure, Sigma-Aldrich) and sodium citrate (99% pure, Sigma-Aldrich). Commercial standards were used for the quantification of cello-oligosaccharides and xylo-oligosaccharides (90%–95% pure, Megazyme)

### 2.2 Biomass and Pretreatment Using Ionic Liquids

The biomass (sugarcane straw) was kindly provided by Brazilian Biorenewables National Laboratory (LNBR) and came from Usina Ferrari (Pirassununga, São Paulo State, Brazil; 21.84309° S, 47.36021° W). The chemical composition of the raw sugarcane straw was 38.68% cellulose, 31.61% hemicellulose, and 20.32% lignin, and it was subjected to pretreatment with a mixture of protic ionic liquids. The variables analyzed in the sugarcane straw pretreatment were temperature, water content, and mixture of the protic ionic liquids 2-hydroxyethylammonium acetate ([Mea]­[Ac]) and 2-hydroxyethylammonium hexanoate (Table 2) according to a study developed by Gonçalves and collaborators.^5^ Protic ionic liquids were synthesized via an acid–base neutralization method, in which a proton (H^+^) is transferred to an NH_2_ group, generating a positively charged species (NH_3_^+^). Temperature was assessed in the range of 75–145 °C, water content from 10 to 60% (w/w), and [Mea]­[Hex] proportion from 5 to 25% (w/w) in the experimental design.^5^ The pretreatment experiments were carried out in a jacketed, stainless steel stirred-tank batch reactor, with a maximum pressure of 10 bar and a working volume of 1 L. All experiments were performed using 36.92 g of straw (15% solid loading) with a moisture content of 7.6% (w/w) and mechanical stirring (3 rpm). After a reaction time of 3 h, the biomass was removed from the reactor and filtered through a 125 μm nylon filter to separate the solid and liquid (liquor) fractions. The solid fraction was washed three times with 300 g of distilled water until neutral pH was reached and then dried in an oven at 105 °C overnight.^5^

**Table 2:** Experimental Design Matrix Varying Temperature, Water Content, and Mixture of Ionic Liquids, Monoethanolamine Acetate (IL_1_) and Monoethanolamine Hexanoate (IL_2_), and Total Release of Cello-Oligosaccharides (COS) and Xylo-Oligosaccharides (XOS) from Pretreated Sugarcane Straw^a^

run	*T* (°C)	water content (%, w w^–1^)	IL_2_ (%, w w^–1^)	COS release (mg g_biomass_^–1^)	XOS release (mg g_biomass_^–1^)
1	–1 (89)	–1 (20)	–1 (9)	4.96	30.81
2	1 (131)	–1 (20)	–1 (9)	4.91	30.51
3	–1 (89)	1 (50)	–1 (9)	2.55	25.31
4	1 (131)	1 (50)	–1 (9)	4.70	31.33
5	–1 (89)	–1 (20)	1 (21)	5.13	28.91
6	1 (131)	–1 (20)	1 (21)	4.68	28.57
7	–1 (89)	1 (50)	1 (21)	2.06	19.15
8	1 (131)	1 (50)	1 (21)	4.12	27.32
9	–1.68 (75)	0 (35)	0 (15)	4.48	29.72
10	1.68 (145)	0 (35)	0 (15)	4.39	30.36
11	0 (110)	–1.68 (10)	0 (15)	4.89	31.71
12	0 (110)	1.68 (60)	0 (15)	2.52	26.18
13	0 (110)	0 (35)	–1.68 (5)	1.67	22.04
14	0 (110)	0 (35)	1.68 (25)	2.04	24.34
CP	0 (110)	0 (35)	0 (15)	4.59	31.62
CP	0 (110)	0 (35)	0 (15)	4.86	31.19
CP	0 (110)	0 (35)	0 (15)	4.51	30.88
CPm	0 (110)	0 (35)	0 (15)	4.65	31.23

a CP, center point; CPm, mean of center-point runs.

The authors found that pretreating the straw with the mixture of protic ionic liquids delignified and deacetylated the biomass in a single step, promoting the removal of inhibitors from the biomass to be used for obtaining oligosaccharides and reducing the number of processing steps, making the process more efficient and optimized.^5^ According to the COS and XOS release, the biomass was selected for the next stage of enzymatic hydrolysis (subsection Production of COS and XOS Separately and Simultaneously through Experimental Design).

#### 2.2.1 Enzymatic Activity

Novozym 50,013 (cellulase-dominant enzyme cocktail) and Shearzyme 500 L (xylanase-dominant enzyme cocktail) enzymatic cocktails were used. Saturation curves were generated to evaluate the reaction rate at a constant level, and sugarcane straw biomass pretreated with ionic liquids of the central points was used as the substrate (5% m v^–1^). After determining the biomass selection (first stage), the enzymatic load was determined from the enzymatic saturation using the selected biomass as the substrate, and the selected biomass was used in the experimental design (second stage). Reactions occurred in test tubes put in a water bath at 50 °C containing biomass (5% m v^–1^), sodium citrate buffer pH 5.0 (50 mM), and different volumes of cellulase (5, 10, 15, 20, 25, 50, 60, 80, and 100 μL) and xylanase (5, 10, 25, 50, 100, 120, 150, 200, 220, and 250 μL), and reaction time of 60 and 30 min, respectively.^22^ Reducing sugars were evaluated using dinitrosalicylic acid (DNS).^23^ The initial enzymatic activity was 90 FPU g_biomass_^–1^ for cellulase (corresponding to 5 μL of enzyme), and enzyme saturation resulted in 250 FPU g_biomass_^–1^ (20 μL of enzyme); the initial enzymatic activity for xylanase was 50 U g_biomass_^–1^ (corresponding to 5 μL of enzyme), and saturation was achieved at 135 U g_biomass_^–1^ (100 μL of enzyme) (Figures S1 and S2, Supporting Information).

### 2.3 Production of COS and XOS Separately and Simultaneously through Experimental Design

First, enzymatic hydrolysis was performed to release COS and XOS separately to select the pretreated biomass (first stage) using the enzymatic load of 10 FPU g_biomass_^–1^ for cellulase and xylanase, separately. Assays were performed in 2 mL tubes (reaction volume of 1 mL) in sodium citrate buffer pH 5.0 (50 mM) and 5.0% (m v^–1^) biomass, temperature of 50 °C for 48 h, and agitation of 1500 rpm in a thermoblock (Loccus Biotecnologia, DB-HS, Brazil).^5^

After biomass selection, the selected biomass was used in experimental design (second stage). A Rotational Central Composite Design (RCCD) 2^3^ was used to investigate the synergistic effects of enzymes on the simultaneous release of COS and XOS.^24^ RCCD consisted of 14 assays and 4 central points (evaluation of process repeatability), totaling 18 assays to evaluate the variables, temperature (35 to 65 °C) and cellulase (0 to 250 FPU g_biom_^–1^), and xylanase (0 to 135 U g_biom_^–1^) enzyme loads. The assays were performed in sodium citrate buffer pH 5.0 and 5.0% (m v^–1^) using the biomass selected in stage 1, at different temperatures and enzymatic loads (Table 3), with stirring at 1500 rpm in a thermoblock (Loccus Biotecnologia, DB-HS, Brazil). Simultaneous release of COS and XOS was evaluated at different times (12, 24, and 48 h). The control reactions were conducted using the maximum load of cellulase (250 FPU g_biom_^–1^) and xylanase (135 U g_biom_^–1^), separately, at different times, at 50 °C under stirring (1500 rpm). The levels of the variables were determined, aiming for the best responses and to reduce costs combined with process time.

**Table 3:** Experimental Design Matrix Varying Temperature and Cellulase and Xylanase Enzyme Loads Simultaneously, Resulting in the Release of Total Cello-Oligosaccharides and Xylo-Oligosaccharides at Different Enzymatic Hydrolysis Times^a^

run	*T* (°C)	cellulase loading (FPU g_biom_^–1^)	xylanase loading (U g_biom_^–1^)	COS release (mg g_biomass_^–1^)			XOS release (mg g_biomass_^–1^)		
				12 h	24 h	48 h	12 h	24 h	48 h
1	–1 (41)	–1 (50.6)	–1 (27.3)	88.85	76.06	56.67	19.80	18.36	17.29
2	1 (59)	–1 (50.6)	–1 (27.3)	80.70	66.59	62.23	15.12	14.55	15.33
3	–1 (41)	1 (199.4)	–1 (27.3)	37.22	20.27	10.89	19.88	19.36	16.67
4	1 (59)	1 (199.4)	–1 (27.3)	54.09	58.17	53.24	13.02	13.20	14.23
5	–1 (41)	–1 (50.6)	1 (107.7)	85.42	74.86	48.15	18.43	19.81	19.35
6	1 (59)	–1 (50.6)	1 (107.7)	87.13	65.78	61.87	16.19	13.11	13.95
7	–1 (41)	1 (199.4)	1 (107.7)	33.47	20.57	11.84	18.32	19.98	15.80
8	1 (59)	1 (199.4)	1 (107.7)	57.74	51.32	53.67	12.75	10.51	12.77
9	–1.68 (35)	0 (125)	0 (67.5)	21.62	13.90	5.06	21.40	18.53	12.53
10	1.68 (65)	0 (125)	0 (67.5)	53.95	52.68	68.49	13.25	10.51	13.89
11	0 (50)	–1.68 (0)	0 (67.5)	44.63	45.57	55.79	9.04	9.59	10.5
12	0 (50)	1.68 (250)	0 (67.5)	66.72	46.72	36.46	19.70	12.29	12.63
13	0 (50)	0 (125)	–1.68 (0)	61.50	47.10	52.04	5.49	5.17	5.56
14	0 (50)	0 (125)	1.68 (135)	73.57	50.16	78.49	17.36	14.82	9.09
CP	0 (50)	0 (125)	0 (67.5)	70.92	61.26	55.97	17.00	19.02	14.71
CP	0 (50)	0 (125)	0 (67.5)	70.86	56.41	53.77	17.95	13.02	14.08
CP	0 (50)	0 (125)	0 (67.5)	74.20	55.33	49.41	18.23	15.05	12.55
CP	0 (50)	0 (125)	0 (67.5)	71.79	58.69	44.04	18.34	13.33	11.98
CPm	0 (50)	0 (125)	0 (67.5)	71.94 ± 1.56	57.92 ± 2.63	50.80 ± 5.27	17.88 ± 0.61	15.11 ± 2.76	13.33 ± 1.28

a CP, center point; CPm, mean of center-point runs.

Validation experiments were conducted using the optimized variables from the experimental design responses. A kinetic study was built at 3, 6, 12, and 18 h. For all enzymatic hydrolysis, at the end of the process time, the tubes were subjected to 99 °C for 5 min for enzyme inactivation, centrifuged at 9000*g* (Eppendorf, AG, Germany), and the supernatant was collected and filtered on a 0.22 μm membrane for subsequent chromatography analysis.^5^

### 2.4 Analytical Methods

COS and XOS were quantified by HPLC-PAD using a Dionex DX-500 system (Sunnyvale, CA, USA), CarboPac PA1 precolumn (4 × 50 mm), and CarboPac PA1 column (4 × 250 mm) at 25 °C and with pulsed amperometric detection. Component separation used gradient A (100 mM NaOH) and B (100 mM NaOH +300 mM NaOAc), injection volume of 25 μL, flow rate of 1 mL min^–1^, and a running time of 25 min^5^. Calibration curves were plotted based on the commercial standards, cellobiose (C2), cellotriose (C3), cellotetraose (C4), cellopentaose (C5), and cellohexaose (C6) for cello-oligosaccharides; xylobiose (X2), xylotriose (X3), xylotetraose (X4), xylopentaose (X5), and xylohexaose (X6) for xylo-oligosaccharides (Megazyme, Bray, Ireland). No coelution was observed between the components in the chromatographic analysis, allowing the individual identification of each component.

### 2.5 Statistical Analysis

Experimental design results for both COS and XOS at different times were analyzed at a 90% confidence level using Protimiza Experimental Design (https://experimental-design.protimiza.com.br/) software. Experiments to determine the saturation curve and validation of the experimental design were conducted in triplicate, whereas the enzymatic hydrolysis for biomass selection was determined in duplicate. The results were presented as mean and standard deviation.

## 3 Results and Discussion

First, the selection of pretreated biomass (sugarcane straw) was defined based on the results of total COS and XOS obtained separately (Table 2). Total release was 4.96 mgCOS g_biomass_^–1^ and 30.81 mgXOS g_biomass_^–1^ under pretreatment conditions at 89 °C, 20% water (w w^–1^), and 91% monoethanolamine acetate (w w^–1^; [Mea]­[Ac]) and 9% monoethanolamine hexanoate (w w^–1^; [Mea]­[Hex]). The chemical composition of biomass run 1 after pretreatment was 47.60% cellulose, 38.41% hemicellulose, and 14.02% lignin (an increase of 19.2% in cellulose and 17.7% in hemicellulose compared to raw sugarcane straw).^5^

Run 1 was selected as the most promising condition for the simultaneous release of COS (4.96 mg g_biom_^–1^) and XOS (30.81 mg g_biom_^–1^), as it uses 57% less hexanoate compared to Run 5, and a lower temperature (19%) and hexanoate concentration (40%), as well as a higher water content (50%) compared to Run 11. Pretreatment with protic ionic liquids promotes the disruption of hydrogen bonds and lignin–hemicellulose interactions, facilitating lignin depolymerization and acting similarly to delignification and deacetylation processes. These processes remove lignin—thereby reducing nonproductive enzyme adsorption—and acetyl groups from hemicellulose, decreasing steric hindrance and increasing substrate hydrophilicity. As a result, significant structural modifications occur, including reduced cellulose crystallinity, increased porosity, and greater exposure of surface area and cellulose microfibrils, ultimately enhancing enzyme accessibility to glycosidic bonds. Additionally, increasing the water content lowers the system viscosity, improving the solubilization of biomass components and reducing the overall ionic liquid demand. Although higher temperatures further enhance the cellulose exposure and promote structural changes such as alterations in crystallinity, oxidation, and cleavage of intermolecular bonds, milder conditions are operationally advantageous, as they reduce energy consumption and lower costs associated with heating and pressurization.^5^ Thus, more biomass can be destined for enzymatic hydrolysis instead of being used for energy generation through burning, in addition to reducing process cost due to the low temperature and the global reduction in ionic liquid usage resulting from the increased water content in the solution.

After the first stage, the pretreated biomass was used as a substrate to conduct the experimental design using the enzyme mixture at different loads and temperatures (Table 3). Experimental design is used to structure, optimize, and understand processes, allowing experiments to be executed efficiently. It is used to identify and evaluate the variables that influence a process, minimize the number of experiments needed to obtain reliable results, and determine how different variables interact with each other and how these interactions affect the final outcome.^5,24–29^ The experimental design evaluated the total release of COS and XOS simultaneously at 12, 24, and 48 h of enzymatic hydrolysis. The variables were statistically analyzed with a confidence level of 90% (*p* < 0.10, confidence level commonly used in biological processes) (Protimiza Experimental Design—https://experimental-design.protimiza.com.br). The total release of COS (5.06–88.85 mg g_biomass_^–1^) and XOS (5.17–21.40 mg g_biomass_^–1^) varied according to the conditions and different enzymatic hydrolysis times evaluated (Table 3).

Effect analysis found that the studied variables presented no statistical significance (*p* < 0.10), thus establishing a model for the responses obtained was infeasible (Tables S1 and S5, Supporting Information). This behavior is associated with the high heterogeneity of raw biomass, which consists of the cellulose–hemicellulose–lignin complex. In addition, the use of different enzymes and enzyme loadings may promote enzyme–substrate competition and involves distinct reaction kinetics, contributing to increased data variability and reduced correlation with the evaluated factors. However, the variables influenced the COS and XOS results. Moreover, the low variation in the repetitions of the center points (15, 16, 17, and 18; Table 3) demonstrated good repeatability of the enzymatic hydrolysis process. Thus, an experimental design was used as a strategy to achieve process improvement by evaluating the interactions between enzyme mixtures, different temperatures, and enzymatic hydrolysis times.

Overall, a reduction in cello-oligosaccharide production (Figure 1) was observed over time due to the prolonged enzymatic hydrolysis time, reducing the monomer. Results of xylo-oligosaccharides (Figure 2) over time were similar to each other, possibly because cellulase may have inhibited the enzymatic action of xylanase or xylanase absorption occurred in the biomass, preventing its action.

**Figure 1:**
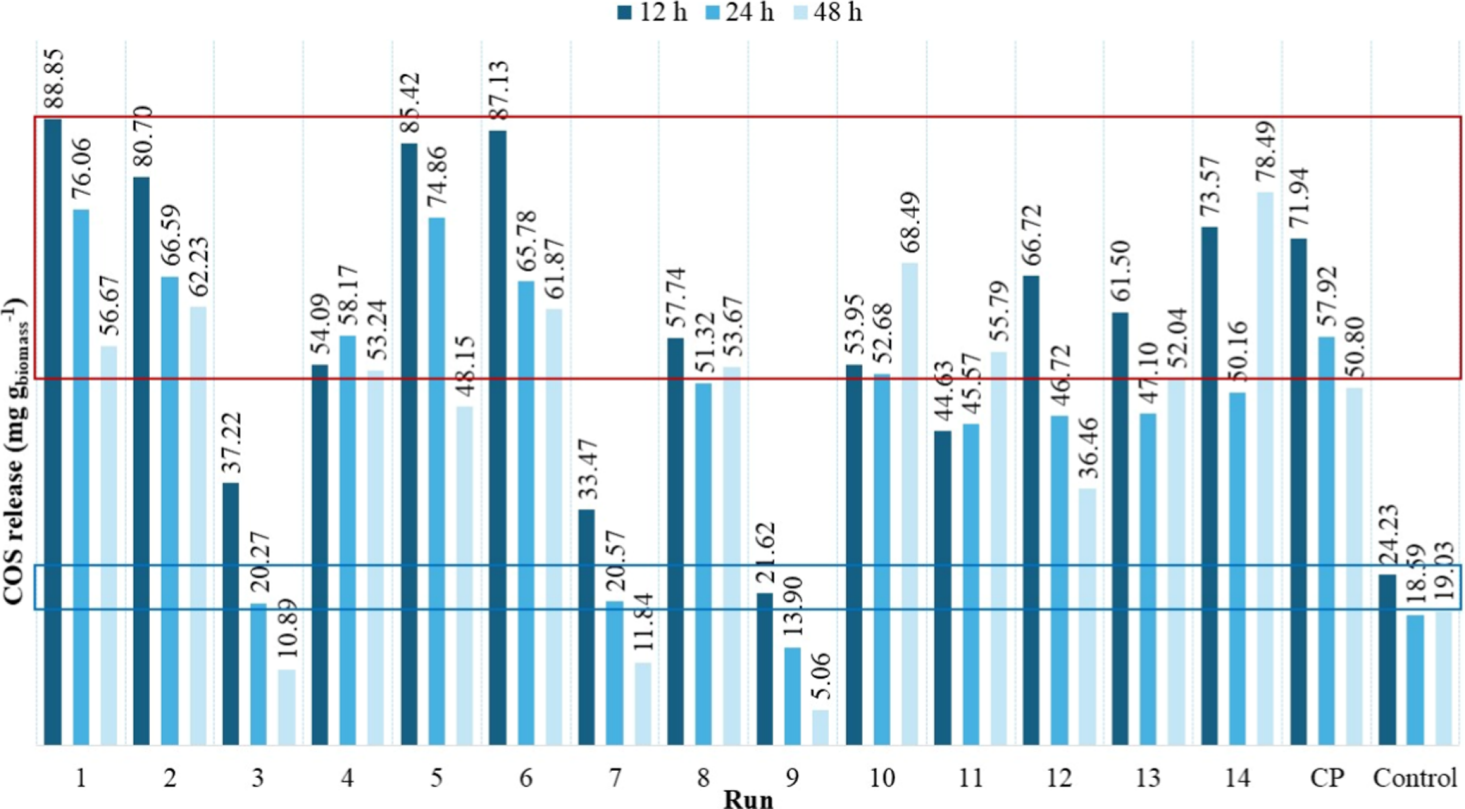
Release of cello-oligosaccharides (COS) at 12, 24, and 48 h using enzymatic mixtures. Red represents the highest results, and blue represents the lowest results from the enzyme mixtures.

**Figure 2:**
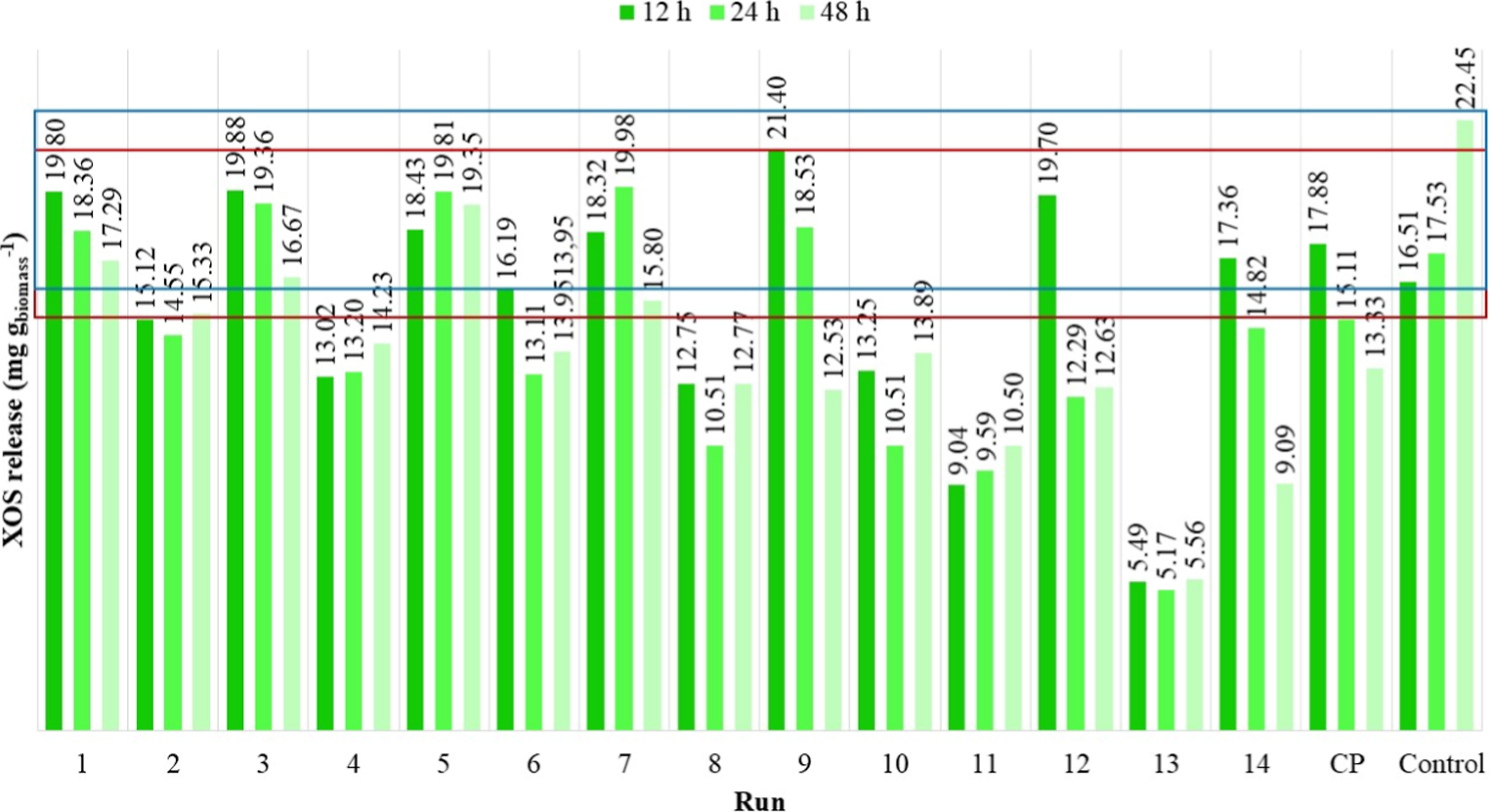
Release of xylo-oligosaccharides (COS) at 12, 24, and 48 h using enzymatic mixtures. Red represents the highest results, and blue represents the lowest results from the enzyme mixtures.

Results of the experimental design showed that run 1 (Table 3) resulted in 88.85 mg g_biomass_^–1^ of total COS released at 12 h of enzymatic hydrolysis using 50.6 FPU g_biom_^–1^ cellulase, 27.3 U g_biom_^–1^ xylanase, and temperature of 41 °C (3.67 times higher compared with control: 24.23 mgCOS g_biomass_^–1^ at 12 h), whereas total XOS was ∼19 mg g_biomass_^–1^ throughout the different times. It was observed that reducing enzyme loads resulted in interesting responses in obtaining oligosaccharides, compared with runs 6 and 7. COS results at 12 h were similar to that of runs 5 and 6 (85.42 mg g_biomass_^–1^ and 87.13 mg g_biomass_^–1^, respectively), but a higher xylanase load (107.7 U g_biom_^–1^) was used. XOS results were also similar, reinforcing the hypothesis that xylanase was adsorbed in the biomass. Barbosa et al. explored COS release from sugarcane straw pretreated hydrothermally and with ionic liquids subjected to hydrolysis by Celluclast 1.5 L using 10 FPU g_biomass_^–1^ at 50 °C for 48 h, resulting in the release of 26.54 mg g_biomass_^–1^ and 85.85 mg g_biomass_^–1^, respectively.^4^ However, the maximum result achieved for COS was under more aggressive pretreatment conditions with ionic liquids (130 °C, 75% [Mea]­[Ac]/25% [Mea]­[Hex] and 20% H_2_O) compared with run 1 and higher temperature and enzymatic hydrolysis time.

Ávila and Goldbeck explored COS production from purified cellulose obtained from sugarcane straw and coffee husks under alkaline hydrothermal pretreatment and a mixture of 3 enzymes purified at 50 °C for 48 h, resulting in 63.56 and 41.78 mgCOS g_substrate_^–1^, respectively.^1^ Gonçalves, Teixeira, and Forte also studied COS release from sugarcane straw subjected to ionic liquids (110 °C, 85% [Mea]­[Ac]/15% [Mea]­[Hex], and 35% H_2_O), evaluating different enzymatic loads of Celluclast 1.5L and temperatures combined with enzymatic kinetics, reaching 99.81 mg g_biomass_^–1^ at 6 h, 46 °C, and 5 FPU g_biomass_^–1^.^5^ These findings reinforce that pretreatment with ionic liquids has advantages in cello-oligosaccharide release from sugar cane straw.

XOS release over time was similar to control, ∼19 mgXOS g_biomass_^–1^ for run 1 (Table 3; Figure 2). Runs 5, 7, and central points resulted in close values using higher xylanase enzyme load compared with run 1, suggesting enzyme adsorption occurred in the biomass, preventing its action. During enzymatic hydrolysis, oligosaccharides and monomers may be generated, which can lead to product inhibition of the enzyme.^30^ Cellobiose, for instance, can interfere with the formation of the cellulase enzyme–substrate complex, thereby preventing or delaying enzymatic activity; a similar inhibitory effect may also have occurred for xylanase.^13,31^ Another important hypothesis that should be highlighted is the presence of residual lignin in the pretreated biomass. Lignin exhibits a unique cross-linked aromatic structure, rich in active functional groups such as phenolic hydroxyls and carboxyl groups, which contribute to lignin interactions with the active sites of certain enzymes, resulting in the inhibition of their activities.^32–34^ XOS production was limited and remained similar throughout the processes. More in-depth studies may be conducted, such as multistage hydrolysis, as well as strategies involving adsorption–desorption mechanisms or the use of surfactants, which may facilitate enzyme–substrate interactions.

Several studies purify hemicellulose from lignocellulosic biomasses to achieve high XOS values. Paschoa et al. extracted xylan from sugarcane bagasse by alkaline treatment, obtaining 225 mg XOS g_substrate_^–1^ using 40 U g_substrate_^–1^ of Shearzyme for 24 h at 50 °C;^16^ and XOS were applied in the development of a carrageenan-based emulsion gel, with potential application in food products.^35^ Silva et al. used bamboo culm as a raw material for XOS production. Hemicellulose was extracted by alkaline solution and subsequently submitted to Shearzyme enzymatic cocktail for 24 h at 50 °C, yielding 250 mg XOS g_substrate_^–1^.^12^ Despite the high XOS results achieved by these studies, the hemicellulosic fraction underwent a purification stage with greater enzymatic hydrolysis of this component. In our study, we used crude biomass after pretreatment, reducing steps to obtain the bioproduct, and shorter enzymatic hydrolysis times (12 h). According to the experimental design results, the presence of xylanase is crucial to achieve high COS results, demonstrating the synergistic action of the two enzymes studied.

Temperature affects the enzymatic hydrolysis process and, according to the results obtained, milder temperatures (41 °C) reached high COS concentrations (runs 1, 5, and CP) combined with lower enzymatic loads. Milder temperatures may have decreased the enzymatic reaction speed, thus favoring the production of cello-oligosaccharides. We thus found a balance between enzymatic load and temperature to obtain this bioproduct from sugarcane straw pretreated with ionic liquids.

The analysis of the degree of polymerization of oligosaccharides is essential to elucidate the selectivity of the enzymatic process, as the oligomer chain length strongly influences biological functionality, fermentability, and application potential. Although the total oligomer yield provides a general measure of process efficiency, the DP profile reveals key aspects of enzymatic action and enables process optimization to obtain specific oligosaccharide fractions, thereby directing product functionality, particularly for food, nutritional, biomedical, and biotechnological applications.^36,37^

Table 4 presents the distribution of short-chain COS (C2–C6) obtained under different experimental conditions over three reaction times. Overall, reaction time exerted a strong influence on both the total COS production and the degree of polymerization distribution, with higher total COS yields observed at 12 h in most runs, indicating greater initial hydrolysis efficiency. At 24 and 48 h, a reduction in total COS was observed, suggesting degradation to monomers, rearrangements in the oligomeric profile, or enzymatic limitations over time. Cellobiose (C2) was the predominant component at shorter reaction times, indicating a more selective and controlled hydrolysis, whereas a decrease in the C2 fraction at longer times suggests its conversion to the corresponding monomer. The formation of higher oligomers (C4–C6) was highlighted by the predominance of C6 at longer reaction times, particularly at 24 and 48 h (e.g., runs 2, 4, 6, and 8), and was influenced by temperature, as higher temperatures were applied. Moreover, the presence of xylanase and the absence of cellulase (run 11) favored the formation of the C6 fraction without the generation of lower-degree oligomers. This behavior may be associated with hemicellulose cleavage and the consequent release of longer chains from the cellulosic fraction of the biomass. This interpretation is supported by the detection of X2, X4, and X6 xylo-oligosaccharide fractions, evidencing the action of xylanase on the hemicellulosic fraction (Table S7, Supporting Information).

**Table 4:** Cello-Oligosaccharide (C2–C6) Profiles as a Function of Reaction Time for Different Experimental Runs According to the Experimental Design^a^

run	time (h)	C2 (mg g_biom_^–1^)	C3 (mg g_biom_^–1^)	C4 (mg g_biom_^–1^)	C5 (mg g_biom_^–1^)	C6 (mg g_biom_^–1^)
1	12	46.05	1.61	1.18	2.53	37.48
	24	43.71	1.26	0.16	0.82	30.19
	48	31.06	1.60	0.31	1.19	22.52
2	12	30.29	1.14	0.04	0.90	48.33
	24	13.75	1.12	0.40	0.94	50.38
	48	7.91	0.94	0.24	0.91	52.23
3	12	31.09	1.53	0.25	0.59	3.75
	24	16.62	1.44	0.00	0.46	1.74
	48	7.96	1.26	0.00	0.15	1.51
4	12	5.94	0.84	0.58	0.43	46.31
	24	2.50	1.07	0.89	0.17	53.54
	48	0.95	0.44	0.65	0.02	51.18
5	12	48.23	1.61	0.21	1.71	33.66
	24	42.67	1.37	0.12	1.90	28.80
	48	28.61	1.56	0.65	0.92	16.39
6	12	31.18	1.44	0.05	2.43	52.03
	24	16.04	1.00	0.52	0.27	47.95
	48	7.22	1.37	0.12	1.02	52.15
7	12	27.98	1.55	0.31	0.55	3.07
	24	16.46	1.69	0.17	0.31	1.93
	48	8.85	1.47	0.08	0.01	1.43
8	12	6.17	1.20	0.86	0.29	49.22
	24	2.28	0.74	0.44	0.00	47.87
	48	1.73	0.38	0.72	0.07	50.77
9	12	19.61	1.75	0.15	0.33	0.05
	24	11.46	1.67	0.17	0.60	0.00
	48	3.65	1.30	0.00	0.11	0.00
10	12	4.05	1.12	0.41	0.92	47.45
	24	3.15	0.95	0.66	0.89	47.03
	48	5.29	1.01	0.93	1.06	47.59
11	12	0.12	0.25	0.00	3.66	40.59
	24	0.00	0.46	0.05	0.04	45.02
	48	0.00	1.00	0.08	3.21	51.49
12	12	15.85	1.81	1.32	0.94	46.79
	24	4.52	1.24	0.47	0.00	40.48
	48	2.13	0.80	0.22	0.00	33.31
13	12	18.70	0.00	2.66	28.19	11.96
	24	7.12	0.00	2.98	25.37	11.63
	48	3.89	0.00	3.89	28.74	15.52
14	12	22.89	1.79	1.19	1.15	46.54
	24	10.77	1.18	0.30	0.07	37.84
	48	4.99	22.76	0.40	0.13	28.21
CP	12	23.16	1.42	0.76	0.55	45.03
	24	13.27	1.33	0.61	0.36	45.69
	48	5.97	1.76	1.02	0.23	46.98
CP	12	24.41	1.19	0.81	0.67	43.78
	24	9.84	1.27	0.95	0.45	43.90
	48	4.98	1.46	0.87	0.14	46.32
CP	12	25.77	1.41	0.94	0.71	45.38
	24	10.62	1.25	0.72	0.46	42.27
	48	4.70	1.55	0.91	0.19	42.07
CP	12	23.39	1.39	1.02	0.85	45.15
	24	12.52	1.24	0.50	0.11	44.33
	48	3.62	1.05	0.38	1.78	37.21
CPm	12	24.18 ± 1.19	1.35 ± 0.11	0.88 ± 0.12	0.69 ± 0.12	44.84 ± 0.72
	24	11.56 ± 1.60	1.27 ± 0.04	0.69 ± 0.19	0.35 ± 0.16	44.05 ± 1.41
	48	4.43 ± 0.97	1.35 ± 0.30	0.72 ± 0.29	0.70 ± 0.80	41.87 ± 4.52

a C2 (cellobiose); C3 (cellotriose); C4 (cellotetraose); C5 (cellopentaose); C6 (cellohexaose); CP, center point; CPm, mean of center point runs.

Cello-oligosaccharides (C2–C6) were obtained from lignocellulosic biomass through a multistage enzymatic hydrolysis process and investigated for their prebiotic potential. Probiotic bacteria (Lactobacillus and Bifidobacterium) demonstrated that COS, particularly cellobiose, were metabolized, promoting cell growth and the production of beneficial metabolites. In addition, the study indicated that COS exhibited good stability under simulated gastrointestinal digestion, reinforcing their potential as prebiotic ingredients.^13^ However, this approach involved multiple processing stages, which may result in increased costs and longer processing times. In the present study, the simultaneous release of COS and XOS from ionic liquid-pretreated biomass was evaluated, and these oligosaccharides may be further explored in biotechnological processes, with potential applications in the food, pharmaceutical, and biotechnology sectors.

As reported above, a satisfactory model was not obtained for the different enzymatic loads, temperatures, and hydrolysis times. As such, an experimental validation test at 40 °C, 50 FPU g_biom_^–1^ cellulase, and 30 U g_biom_^–1^ xylanase (values within the study ranges) was conducted at 3 h, 6 h, 12 h, and 18 h of enzymatic hydrolysis (Table 5). Productivity is a key parameter as a measure of efficiency, allowing us to evaluate and monitor the performance of our enzymatic process. COS and XOS values were similar, and productivity was reduced over time, showing that shorter hydrolysis times are efficient to achieve the maximum release of bioproducts. The maximum release of COS 83.07 mg g_Biomass_^–1^ was achieved at 18 h (Figure 3), corresponding to a productivity of 4.62 mgCOS g_Biomass_^–1^ h^–1^ and specific productivity of 0.09 mg COS FPU^–1^ h^–1^. The maximum release of XOS achieved was 20.59 mg g_Biomass_^–1^ at 18 h, productivity of 1.14 mg XOS g_Biomass_^–1^ h^–1^, and specific productivity of 0.04 mg XOS U^1–^ h^–1^ (Table 5). The productivity in 3 h of hydrolysis reached 25.89 mg COS g_Biomass_^–1^ h^–1^ (77.66 mg COS g_Biomass_^–1^) and 6.42 mg XOS g_Biomass_^–1^ h^–1^ (19.27 mg XOS g_Biomass_^–1^), i.e., 5.6 times higher (for both COS and XOS) compared to 18 h of process, and showed a decreasing trend with increasing hydrolysis time (Figure 4), confirming that the synergistic process of enzymes and temperature is more efficient at short enzymatic hydrolysis times. The temperature (40 °C) and the enzymatic loads of cellulase (50 FPU g_biom_^–1^) and xylanase (30 U g_biom_^–1^) were reduced, improving the process and achieving the maximum COS and XOS released simultaneously from sugarcane straw pretreated with ionic liquids. Thus, the overall process costs are reduced, providing the simultaneous release of oligosaccharides through the enzyme mixture. Oligosaccharides have several applications in the pharmaceutical, food, and biotechnology industries, and this research allows future studies to apply, purify, or recover these bioproducts using polymeric membranes and/or columns for future applications.^1,13,38^

**Table 5:** Concentration and Productivity at Different Times for Simultaneous Obtaining of Total Cell-Oligosaccharides (COS) and Xylo-Oligosaccharides (XOS) from Sugarcane Straw Pretreated with a Mixture of Protic Ionic Liquids^a^

run	*T* (°C)	cellulase loading (FPU gbiom–1)	xylanase loading (U gbiom–1)	3 h	6 h	12 h	18 h	24 h	48 h
				COS | XOS release (mg g_biomass_^–1^)					
1	41	50.6	27.3	-	-	88.85 | 19.80	-	76.06 | 18.36	56.67 | 17.29
CP_m_	50	125	67.5	-	-	71.94 | 17.88	-	57.92 | 15.11	50.80 | 13.33
validation	40	50	30	77.66 | 19.27	81.52 | 19.52	73.45 | 18.76	83.07 | 20.59	-	-
				productivity COS | XOS (mg g_biomass_^–1^ h^–1^)					
1	41	50.6	27.3			7.40 | 1.65		3.17 | 0.77	1.18 | 0.36
CP_m_	50	125	67.5			6.00 | 1.49		2.41 | 0.63	1.06 | 0.28
validation	40	50	30	25.89 | 6.42	13.59 | 3.25	6.12 | 1.56	4.62 | 1.14		
				specific productivity COS | XOS (mg FPU^–1^ h^–1^|U^1–^ h^–1^)					
1	41	50.6	27.3			0.15 | 0.06		0.06 | 0.00	0.02 | 0.00
CP_m_	50	125	67.5			0.05 | 0.02		0.02 | 0.00	0.01 | 0.00
validation	40	50	30	0.52 | 0.21	0.27 | 0.11	0.12 | 0.05	0.09 | 0.04		

a CPm, mean of center point runs.

**Figure 3:**
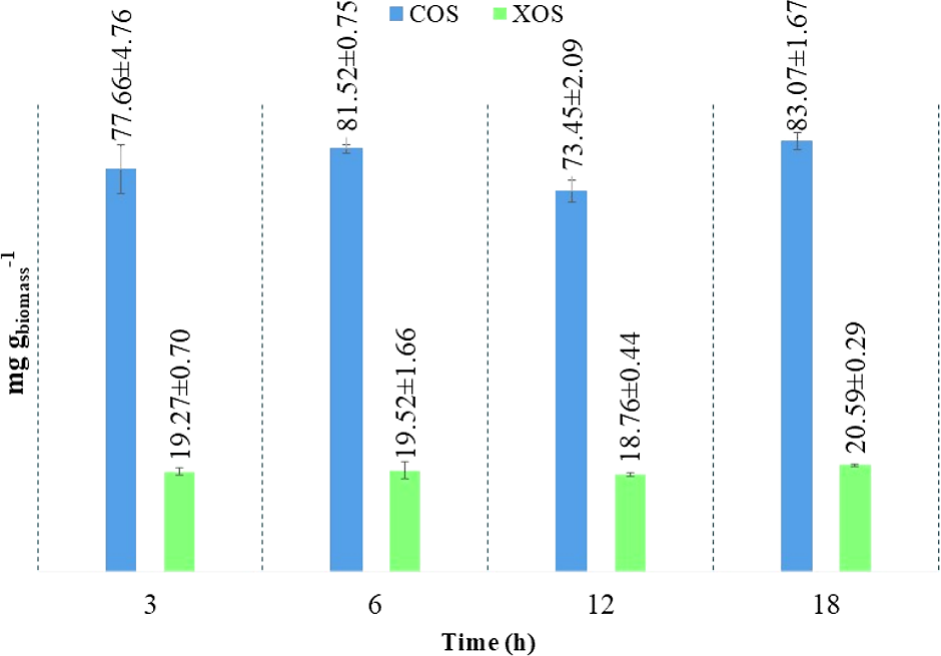
Total cello-oligosaccharides (COS) and xylo-oligosaccharides (XOS) released simultaneously from sugarcane straw under experimental validation conditions (40 °C, 50 FPU g_biom_^–1^ cellulase and 30 U g_biom_^–1^) at different enzymatic hydrolysis times.

**Figure 4:**
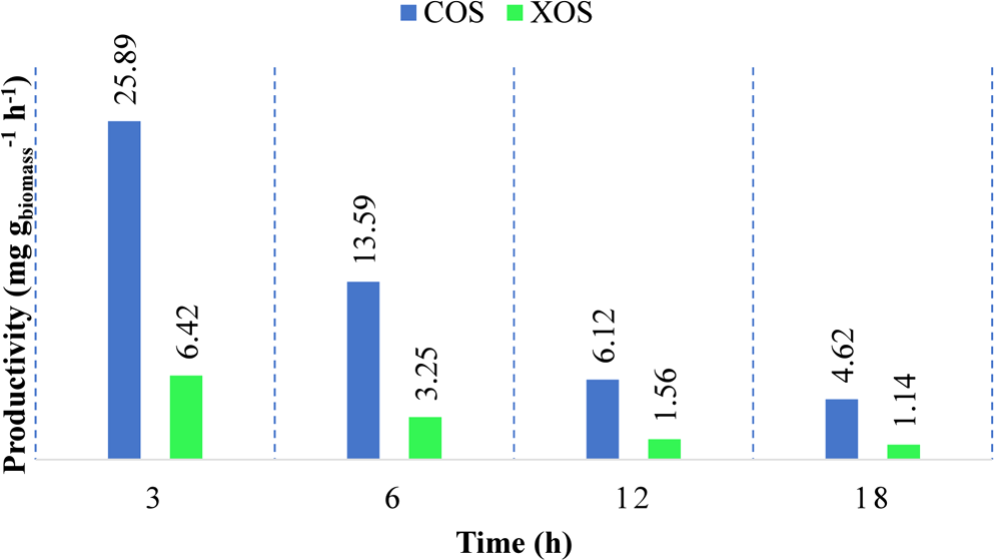
Productivity of cello-oligosaccharides (COS) and xylo-oligosaccharides (XOS) released simultaneously from sugarcane straw under experimental validation conditions (40 °C, 50 FPU g_biom_^–1^ cellulase and 30 U g_biom_^–1^) at different enzymatic hydrolysis times.

Short reaction times favor the formation of COS with a lower degree of polymerization and higher overall yields, whereas prolonged reaction times promote the accumulation of larger oligomers (mainly C6) (Table 6), often at the expense of a reduction in total COS. These results highlight the importance of adjusting the reaction time to modulate the COS profile, as shorter times favor the production of lower-degree-of-polymerization COS and higher total yields, while longer times lead to the accumulation of larger oligomers (particularly C6), accompanied by a decrease in total COS. Reaction time control is therefore a key parameter for selectively directing production, depending on the intended application.

**Table 6:** Profiles of Cello-Oligosaccharides (COS) and Xylo-Oligosaccharides (XOS) Simultaneously Released from Sugarcane Straw under Experimental Validation Conditions at Different Enzymatic Hydrolysis Times

time (h)	C2 | X2 (mg/gbiom)	C3 | X3 (mg/gbiom)	C4 | X4 (mg/gbiom)	C5 | X5 (mg/gbiom)	C6 | X6 (mg/gbiom)
3	37.50 | 4.22	0.90 | 0.00	0.42 | 5.66	0.83 | 1.51	38.01 | 5.27
6	40.28 | 4.12	0.94 | 0.00	0.39 | 8.45	0.86 | 1.82	39.06 | 5.18
12	38.51 | 3.94	0.78 | 0.00	0.12 | 7.99	0.62 | 1.42	33.41 | 5.39
18	44.50 | 4.03	1.12 | 0.00	0.28 | 8.72	1.34 | 2.08	35.81 | 6.02

Several studies based on in vitro and in vivo experiments have suggested that COS exhibit prebiotic properties.^6–11,39^ Among them, cellobiose and cellotriose, which correspond to COS with the lowest degrees of polymerization, stand out as the main substrates supporting the in vitro growth of a representative selection of probiotic bacterial strains.^40^ Low-DP fractions (C2–C3 and X2–X3) are highly water-soluble and rapidly fermented by intestinal microorganisms, such as Bifidobacterium and Lactobacillus, making them particularly effective as prebiotics. These short-chain molecules are preferentially utilized by beneficial bacteria, promoting the production of short-chain fatty acids and contributing to gut health. In addition, their lower molecular weight enhances diffusion and bioavailability, which are desirable characteristics for nutraceutical applications. In contrast, higher-DP fractions (C4–C6 and X4–X6) exhibit lower fermentation rates and greater resistance to degradation, resulting in more prolonged effects in the gastrointestinal tract. These oligosaccharides may contribute to sustained metabolite release and, in some cases, display additional functional properties such as increased viscosity, water retention, and structural stability. These characteristics make them more suitable for applications as food additives, including texturizing agents and stabilizers.^41,42^

More detailed studies can be conducted using the enzymatic hydrolysate obtained from ionic liquid-pretreated sugarcane straw as a prebiotic, since cellobiose is one of the major components generated during enzymatic hydrolysis. In addition, the simultaneous production of COS and XOS offers significant advantages over purified fractions, particularly from the perspectives of process steps, efficiency, and sustainability. This approach enables reductions in cost and processing time by eliminating additional separation and purification steps, while also promoting improved utilization of the cellulosic and hemicellulosic fractions of biomass.

Future studies may evaluate in vitro and in vivo fermentability using different probiotic bacterial consortia to investigate potential synergistic prebiotic effects arising from the combined presence of COSs and XOSs. Furthermore, oligosaccharide mixtures provide greater technological versatility for applications in foods and biotechnological products while contributing to more sustainable processes aligned with the biorefinery concept.

## 4 Conclusion

The present study used a mixture of cellulase and xylanase enzymes for the simultaneous production of cello- and xylo-oligosaccharides, finding that enzyme load and temperature influenced the production of oligosaccharides. COS and XOS were obtained simultaneously (in terms of concentration and productivity) by reducing enzyme load, temperature, and time, favoring the maximum release of the biocomponents. The proposed approach achieved a productivity up to 5.6-fold higher in 3 h compared to enzymatic hydrolysis conducted over 18 h, as well as a 16-fold reduction in processing time relative to conventional hydrolysis performed for 48 h, demonstrating a clear advantage in process intensification.

Additionally, the use of a lower ionic liquid and temperature concentration during pretreatment contributed to enhanced oligosaccharide release while simultaneously reducing the reagent consumption and associated costs. Compared to conventional multistep processes, which typically require fractionation, purification, and multiple processing stages, the proposed one-pot pretreatment strategy combined with the direct use of pretreated raw biomass simplifies the overall process, reduces enzyme and solvent consumption, and improves the operational efficiency.

From an application perspective, these results indicate strong potential for integration into lignocellulosic biorefineries, particularly for the valorization of sugarcane straw into high-value functional oligosaccharides. The combination of reduced processing time, lower energy demand, and decreased reagent consumption aligns with the principles of green chemistry and supports the development of more economically viable and sustainable bioprocesses. This study represents a relevant advancement in biomass conversion by demonstrating a simplified and efficient route for the coproduction of COS and XOS, contributing to the expansion of value-added products within the bioeconomy framework.

## Supplementary Material



## Notes

Experimental data on enzymatic hydrolysis, experimental design, and the production of COS and XOS (PDF)
