# Supplementary material for: Synergic Enzymatic Strategy for Simultaneous Cello-/Xylo-Oligosaccharide Production from Sugarcane Straw via a Novel One-Step Protic Ionic Liquid Delignification–Deacetylation Pretreatment
Source: ACS Omega. 2026 Jul 1;11(27):39653–64. doi: 10.1021/acsomega.5c12305 (PMC13382676; doi:10.1021/acsomega.5c12305)
Supplement: Supplementary file 1 [file ao5c12305_si_001.pdf]

SUPPORTING INFORMATION

Synergic Enzymatic Strategy for Simultaneous Cello/Xylo-Oligosaccharides Production from Sugarcane Straw via a Novel One-Step Protic Ionic Liquid Delignification–Deacetylation Pretreatment

Igor Severo Gonçalves,[a] Rosana Goldbeck Coelho,[a] Telma Teixeira Franco [b] and Marcus Bruno Soares Forte[a,*]

[a] Bioprocess and Metabolic Engineering Laboratory, Department of Food Engineering and Technology, Faculty of Food Engineering, Universidade Estadual de Campinas (UNICAMP), Rua Monteiro Lobato, 80, Zeferino Vaz, Campinas, São Paulo, 13083-862, Brazil.

[b] Faculty of Chemical Engineering, Universidade Estadual de Campinas (UNICAMP), Rua Albert Einstein, 500, Zeferino Vaz, Campinas, São Paulo, 13083-852, Brazil.

*Corresponding author: forte@unicamp.br

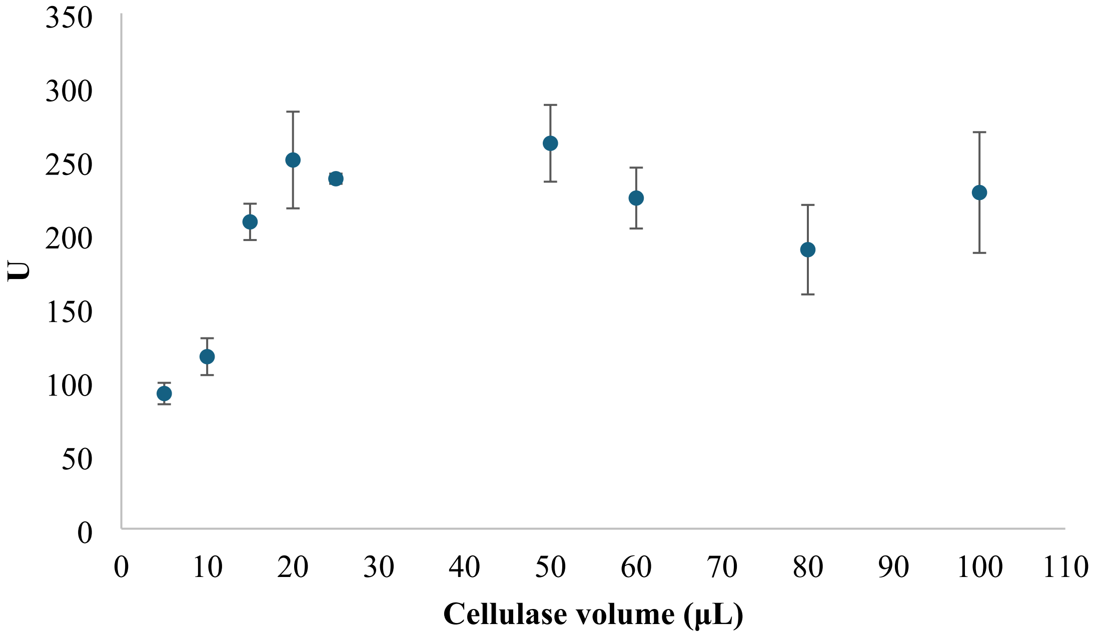

Figure S1. Saturation curve of cellulase Novozym 50013 using sugarcane straw biomass pretreated with ionic liquids as substrate

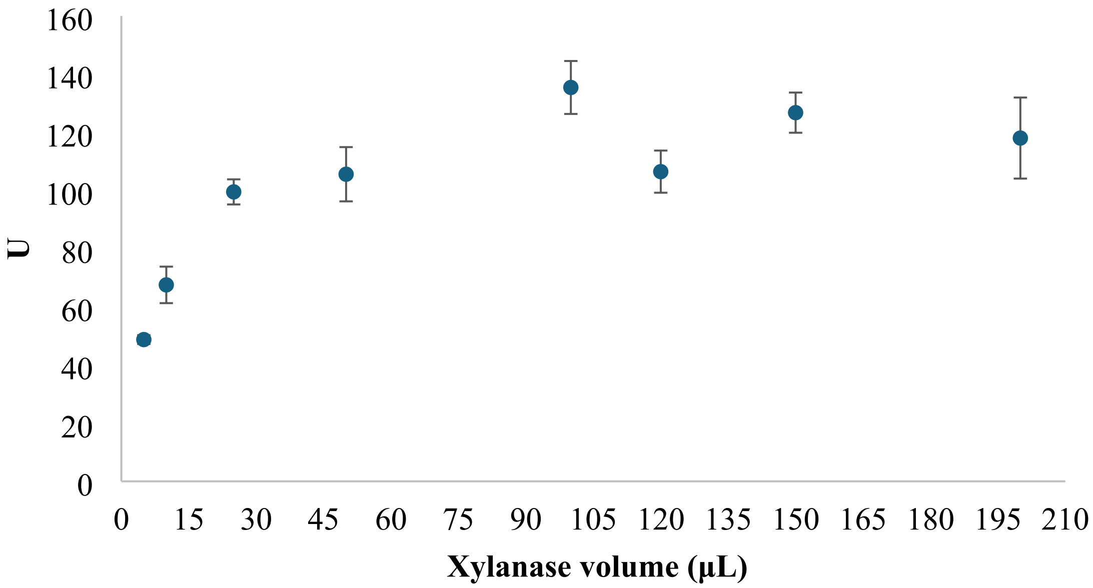

Figure S2. Saturation curve of xylanase Shearzyme 500 L using sugarcane straw biomass pretreated with ionic liquids as substrate

Table S1. Regression analysis of the rotational central composite design responses for release of cello-oligosaccharides (COS) at different enzymatic hydrolysis times using enzymatic mixture.

Factor	Regression coefficient	Standard error	*t*	*p*-value
CCRD 2^3 for cello-oligosaccharide production in 12 h				
Mean	71.31	9.68	7.37	<0.001
(1) Temperature (L)	6.52	5.24	1.24	0.249
Temperature (Q)	−9.26	5.45	−1.70	0.128
(2) Cellulase (L)	−8.96	5.24	−1.71	0.126
Cellulase (Q)	−2.93	5.45	−0.54	0.605
(3) Xylanase (L)	1.70	5.24	0.32	0.755
Xylanase (Q)	1.26	5.45	0.23	0.823
(1) · (2)	5.95	6.85	0.87	0.411
(1) · (3)	2.16	6.85	0.31	0.761
(2) · (3)	−0.39	6.85	−0.06	0.956
R^2 = 52.27%				
CCRD 2^3 for cello-oligosaccharide production in 24 h				
Mean	57.32	7.02	8.17	<0.001
(1) Temperature (L)	8.45	3.80	2.22	0.057
Temperature (Q)	−6.02	3.95	−1.52	0.166
(2) Cellulase (L)	−9.59	3.80	−2.52	0.036
Cellulase (Q)	−1.47	3.95	−0.37	0.719
(3) Xylanase (L)	−0.25	3.80	−0.07	0.949
Xylanase (Q)	−0.59	3.95	−0.15	0.885
(1) · (2)	10.90	4.97	2.19	0.060
(1) · (3)	−0.84	4.97	−0.17	0.869
(2) · (3)	−0.57	4.97	−0.11	0.912
R^2 = 69.83%				
CCRD 2^3 for cello-oligosaccharide production in 48 h				
Mean	51.02	4.59	11.11	<0.001
(1) Temperature (L)	15.39	2.49	6.18	0.000
Temperature (Q)	−5.93	2.59	−2.29	0.051
(2) Cellulase (L)	−9.65	2.49	−3.88	0.005
Cellulase (Q)	−2.62	2.59	−1.01	0.340
(3) Xylanase (L)	2.71	2.49	1.09	0.308
Xylanase (Q)	4.14	2.59	1.60	0.148
(1) · (2)	8.11	3.25	2.49	0.037
(1) · (3)	0.96	3.25	0.29	0.776
(2) · (3)	1.28	3.25	0.39	0.703
R^2 = 89.94%				

Factors significant at $p < 0.10$.

Table S3. Analysis of variance of the responses of the central composite rotatable design for release of cello-oligosaccharides (COS) at different enzymatic hydrolysis times using enzymatic mixture.

**Source of variation**	**Sum of squares**	**Degrees of freedom**	**Mean squares**	$\boldsymbol{F_{calc}}$
CCRD 2^3 for cello-oligosaccharide production in 12 h				
Regression	3291.3	9	365.7	1.0
Residuals	3005.4	8	375.7	
Total	6296.7	17		
$F_{tab(9,8,0.10)} = 2.56$				
CCRD 2^3 for cello-oligosaccharide production in 24 h				
Regression	3656.2	9	406.2	2.1
Residuals	1579.8	8	197.5	
Total	5236.0	17		
$F_{tab(9,8,0.10)} = 2.56$				
CCRD 2^3 for cello-oligosaccharide production in 48 h				
Regression	6054.3	9	672.7	8.0
Residuals	676.9	8	84.6	
Total	6731.2	17		
$F_{tab(9,8,0.10)} = 2.56$				

Table S4. Regression analysis of the rotational central composite design responses for release of xylo-oligosaccharides (XOS) at different enzymatic hydrolysis times using enzymatic mixture.

Factor	Regression coefficient	Standard error	*t*	*p*-value
CCRD 2^3 for xylo-oligosaccharide production in 12 h				
Mean	17.75	1.93	9.22	<0.001
(1) Temperature (L)	−2.42	1.04	−2.32	0.049
Temperature (Q)	0.35	1.08	0.32	0.754
(2) Cellulase (L)	0.91	1.04	0.87	0.411
Cellulase (Q)	−0.69	1.08	−0.64	0.541
(3) Xylanase (L)	1.31	1.04	1.25	0.246
Xylanase (Q)	−1.73	1.08	−1.60	0.149
(1) · (2)	−0.69	1.36	−0.50	0.628
(1) · (3)	0.47	1.36	0.34	0.741
(2) · (3)	−0.19	1.36	−0.14	0.892
R^2 = 58.54%				
CCRD 2^3 for xylo-oligosaccharide production in 24 h				
Mean	14.89	1.99	7.50	<0.001
(1) Temperature (L)	−2.90	1.08	−2.69	0.027
Temperature (Q)	0.76	1.12	0.68	0.514
(2) Cellulase (L)	0.13	1.08	0.12	0.908
Cellulase (Q)	−0.50	1.12	−0.45	0.666
(3) Xylanase (L)	1.04	1.08	0.96	0.364
Xylanase (Q)	−0.84	1.12	−0.75	0.477
(1) · (2)	−0.64	1.41	−0.45	0.662
(1) · (3)	−0.78	1.41	−0.55	0.596
(2) · (3)	−0.26	1.41	−0.18	0.859
R^2 = 56.16%				
CCRD 2^3 for xylo-oligosaccharide production in 48 h				
Mean	13.08	1.90	6.90	<0.001
(1) Temperature (L)	−0.77	1.03	−0.75	0.474
Temperature (Q)	1.07	1.07	1.00	0.346
(2) Cellulase (L)	−0.21	1.03	−0.20	0.843
Cellulase (Q)	0.49	1.07	0.46	0.661
(3) Xylanase (L)	0.31	1.03	0.31	0.768
Xylanase (Q)	−1.01	1.07	−0.95	0.371
(1) · (2)	0.24	1.34	0.18	0.864
(1) · (3)	−0.50	1.34	−0.37	0.718
(2) · (3)	−0.38	1.34	−0.28	0.786
R^2 = 30.69%				

Factors significant at $p < 0.10$.

Table S5. Analysis of variance of the responses of the central composite rotatable design for release of xylo-oligosaccharides (XOS) at different enzymatic hydrolysis times using enzymatic mixture.

**Source of variation**	**Sum of squares**	**Degrees of freedom**	**Mean squares**	$\boldsymbol{F_{calc}}$
CCRD 2^3 for xylo-oligosaccharide production in 12 h				
Regression	168.0	9	18.7	1.3
Residuals	119.0	8	14.9	
Total	287.0	17		
$F_{tab(9,8,0.10)} = 2.56$				
CCRD 2^3 for xylo-oligosaccharide production in 24 h				
Regression	162.2	9	18.0	1.1
Residuals	126.6	8	15.8	
Total	288.8	17		
$F_{tab(9,8,0.10)} = 2.56$				
CCRD 2^3 for xylo-oligosaccharide production in 48 h				
Regression	51.4	9	5.7	0.4
Residuals	115.3	8	14.4	
Total	166.7	17		
$F_{tab(9,8,0.10)} = 2.56$				

Table S6. Productivity and specific productivity of cello- and xylo-oligosaccharides obtained by hydrolysis of sugarcane straw using Novozym 50013 and Shearzyme 500 L for different times.

Run	Productivity (mg $g_{biomassa}^{-1}$)						Specific productivity (mg FPU^{-1} h^{-1} | mg U^{-1} h^{-1})					
	COS			XOS			COS			XOS		
	12 h	24 h	48 h	12 h	24 h	48 h	12 h	24 h	48 h	12 h	24 h	48 h
1	7.40	3.17	1.18	1.65	0.77	0.36	0.15	0.06	0.02	0.06	0.00	0.00
2	6.73	2.77	1.30	1.26	0.61	0.32	0.13	0.05	0.03	0.05	0.00	0.00
3	3.10	0.84	0.23	1.66	0.81	0.35	0.02	0.00	0.00	0.06	0.00	0.00
4	4.51	2.42	1.11	1.09	0.55	0.30	0.02	0.01	0.01	0.04	0.00	0.00
5	7.12	3.12	1.00	1.54	0.83	0.40	0.14	0.06	0.02	0.01	0.00	0.00
6	7.26	2.74	1.29	1.35	0.55	0.29	0.14	0.05	0.03	0.01	0.00	0.00
7	2.79	0.86	0.25	1.53	0.83	0.33	0.01	0.00	0.00	0.01	0.00	0.00
8	4.81	2.14	1.12	1.06	0.44	0.27	0.02	0.01	0.01	0.01	0.00	0.00
9	1.80	0.58	0.11	1.78	0.77	0.26	0.01	0.00	0.00	0.03	0.00	0.00
10	4.50	2.20	1.43	1.10	0.44	0.29	0.04	0.02	0.01	0.02	0.00	0.00
11	3.72	1.90	1.16	0.75	0.40	0.22	0.00	0.00	0.00	0.01	0.00	0.00
12	5.56	1.95	0.76	1.64	0.51	0.26	0.02	0.01	0.00	0.02	0.00	0.00
13	5.13	1.96	1.08	0.46	0.22	0.12	0.04	0.02	0.01	0.00	0.00	0.00
14	6.13	2.09	1.64	1.45	0.62	0.19	0.05	0.02	0.01	0.01	0.00	0.00
CP	5.91	2.55	1.17	1.42	0.79	0.31	0.05	0.02	0.01	0.02	0.00	0.00
CP	5.91	2.35	1.12	1.50	0.54	0.29	0.05	0.02	0.01	0.02	0.00	0.00
CP	6.18	2.31	1.03	1.52	0.63	0.26	0.05	0.02	0.01	0.02	0.00	0.00
CP	5.98	2.45	0.92	1.53	0.56	0.25	0.05	0.02	0.01	0.02	0.00	0.00
Control	2.02	0.77	0.40	1.38	0.73	0.47	0.01	0.00	0.00	0.01	0.00	0.00

Table S7. Xyllo-oligosaccharide (X2–X6) profiles as a function of reaction time for different experimental runs according to the experimental design.

Run	Time (h)	X2 (mg g_{biom}^{-1})	X3 (mg g_{biom}^{-1})	X4 (mg g_{biom}^{-1})	X5 (mg g_{biom}^{-1})	X6 (mg g_{biom}^{-1})
1	12	3.97	0.00	11.99	3.84	6.30
	24	4.07	0.00	12.00	2.29	6.35
	48	4.01	0.00	11.50	1.78	6.13
2	12	3.96	0.00	10.03	1.12	5.36
	24	3.91	0.00	9.94	0.70	5.26
	48	3.96	0.00	11.08	0.29	5.27
3	12	4.18	1.91	12.68	1.11	5.91
	24	4.47	1.92	12.36	0.61	5.86
	48	4.74	0.00	11.72	0.21	6.18
4	12	1.91	0.00	10.65	0.46	5.57
	24	0.00	0.00	12.11	1.09	6.30
	48	0.00	0.00	12.41	1.82	6.44
5	12	4.10	0.00	12.54	1.80	5.75
	24	4.21	0.00	13.16	2.44	6.53
	48	4.69	1.93	11.56	1.17	6.28
6	12	4.10	0.00	10.76	1.33	5.57
	24	3.99	0.00	8.98	0.14	5.02
	48	3.99	0.00	9.62	0.34	5.31
7	12	4.20	1.91	11.59	0.62	5.63
	24	4.14	3.78	11.56	0.49	6.19
	48	5.19	0.00	10.26	0.35	6.03
8	12	1.95	0.00	10.47	0.34	6.06
	24	0.00	0.00	10.08	0.43	6.09
	48	0.00	0.00	11.60	1.18	6.78
9	12	4.28	3.82	9.64	1.66	5.99
	24	4.88	0.00	12.50	1.15	6.31
	48	0.00	0.00	11.71	0.83	6.06
10	12	4.78	0.00	8.13	0.34	4.71
	24	2.42	0.00	7.48	0.61	4.68
	48	3.83	0.00	7.44	0.52	4.72
11	12	4.48	0.00	4.66	0.00	4.14
	24	4.58	0.00	4.92	0.08	4.15
	48	4.64	0.00	4.95	0.92	4.16
12	12	3.91	0.00	14.31	1.48	6.92
	24	0.00	0.00	11.56	0.73	6.51
	48	0.00	0.00	12.16	0.47	6.70
13	12	0.00	0.00	5.05	0.44	5.80
	24	0.00	0.00	5.00	0.17	5.63
	48	0.00	0.00	5.32	0.25	6.21

14	12	4.00	0.00	12.23	1.14	6.42
	24	3.91	0.00	10.52	0.38	6.09
	48	0.00	0.00	8.97	0.12	5.89
CP	12	4.19	0.00	11.58	1.23	6.07
	24	4.09	0.00	14.03	0.90	6.39
	48	0.00	0.00	13.94	0.77	6.63
CP	12	4.26	0.00	12.33	1.35	6.33
	24	0.00	0.00	11.98	1.04	6.39
	48	0.00	0.00	13.38	0.70	6.52
CP	12	4.23	0.00	12.49	1.51	6.19
	24	2.14	0.00	12.02	0.90	6.07
	48	0.00	0.00	11.89	0.66	6.74
CP	12	4.27	0.00	12.57	1.50	6.30
	24	0.00	0.00	12.61	0.72	6.33
	48	0.00	0.00	11.67	0.31	6.14
CPm	12	4.24±0.04	0.00±0.00	12.24±0.45	1.40±0.13	6.22±0.12
	24	1.56±1.97	0.00±0.00	12.66±0.96	0.89±0.13	6.29±0.15
	48	0.00±0.00	0.00±0.00	12.72±1.11	0.61±0.20	6.51±0.26

X2 (xyllobiosis); X3 (xyllotriosis); X4 (xyllotetraose); X5 (xyllopentaose); X6 (xyllohexaose); CP, center point; CPm, mean of center point runs.
